# Effect of depression on phase coherence between respiratory sinus arrhythmia and respiration during sleep in patients with obstructive sleep apnea

**DOI:** 10.3389/fphys.2023.1181750

**Published:** 2023-09-29

**Authors:** Yahya Alzaabi, Ahsan H. Khandoker

**Affiliations:** Healthcare Engineering Innovation Center (HEIC), Department of Biomedical Engineering, Khalifa University of Science and Technology, Abu Dhabi, United Arab Emirates

**Keywords:** obstructive sleep apnea, major depressive disorder, respiratory sinus arrhythmia, autonomic nervous system, ECG, sleep stages

## Abstract

**Introduction:** A high prevalence of major depressive disorder (MDD) among Obstructive Sleep Apnea (OSA) patients has been observed in both community and clinical populations. Due to the overlapping symptoms between both disorders, depression is usually misdiagnosed when correlated with OSA. Phase coherence between respiratory sinus arrhythmia (RSA) and respiration (λ _RSA-RESP_) has been proposed as an alternative measure for assessing vagal activity. Therefore, this study aims to investigate if there is any difference in λ _RSA-RESP_ in OSA patients with and without MDD.

**Methods:** Electrocardiograms (ECG) and breathing signals using overnight polysomnography were collected from 40 OSA subjects with MDD (OSAD+), 40 OSA subjects without MDD (OSAD-), and 38 control subjects (Controls) without MDD and OSA. The interbeat intervals (RRI) and respiratory movement were extracted from 5-min segments of ECG signals with a single apneic event during non-rapid eye movement (NREM) [353 segments] and rapid eye movement (REM) sleep stages [298 segments]. RR intervals (RRI) and respiration were resampled at 10 Hz, and the band passed filtered (0.10–0.4 Hz) before the Hilbert transform was used to extract instantaneous phases of the RSA and respiration. Subsequently, the λ _RSA-RESP_ between RSA and Respiration and Heart Rate Variability (HRV) features were computed.

**Results:** Our results showed that λ _RSA-RESP_ was significantly increased in the OSAD+ group compared to OSAD- group during NREM and REM sleep. This increase was accompanied by a decrease in the low frequency (LF) component of HRV.

**Discussion:** We report that the phase synchronization index between RSA and respiratory movement could provide a useful measure for evaluating depression in OSA patients. Our findings suggest that depression has lowered sympathetic activity when accompanied by OSA, allowing for stronger synchronization between RSA and respiration.

## 1 Introduction

Obstructive sleep apnea (OSA) is defined as frequent episodes of blockages of the upper airway during sleep. It is especially characterized by sleep decreases (hypopnea) or pauses (apnea) in the breathing (Schröder and O’Hara, 2005). The existence and severity of OSA are measured by an apnea-hypopnea index (AHI), representing the number of hypopnea and apnea events per hour of sleep. OSA is often associated with a wide range of comorbidities. Some of the most common comorbid conditions include cardiovascular, respiratory, endocrine, metabolic, and psychiatric symptoms (Ejaz et al., 2011). Many studies have shown a significant association between OSA and the depressive symptomatology (Sivertsen et al., 2008; Ejaz et al., 2011; Rey de Castro and Rosales-Mayor, 2013). More specifically, the prevalence of depression in OSA patients has been reported to be 5%–63% (BaHammam et al., 2016).

The relationship between OSA and depression is complex. Both conditions exhibit overlapping symptoms. It is not clear whether these shared symptoms result in the potential for a clinician to confuse one syndrome with the other, leading to misdiagnosis, or whether one syndrome may increase the likelihood for the other to occur. The most frequently occurring symptoms of OSA and depression are sleep disturbance and fatigue. These issues can disguise each other because of their similarities (Harris et al., 2009). The comorbidity of OSA and depression also might suggest that both illnesses may share a neurobiological pathway. The serotoninergic system, which has a central role in regulating mood and circadian rhythm, also controls the hypoglossal nucleus, which controls the upper-airway dilatator motor neurons (Schröder and O’Hara, 2005).

The autonomic nervous system (ANS) consists of parasympathetic and sympathetic branches. Functionally, the parasympathetic inputs exert inhibitory control on the heart rate via directly innervating the heart’s sinoatrial node by the vagus nerve. The vagus nerve controls the heart by slowing the heart rate during rest to favour the parasympathetic dominance. In contrast, vagal withdrawal occurs during periods of stress or threat, allowing for greater sympathetic activities. According to Polyvagal Theory, the vagal system adapts to social or environmental changes by quickly shifting anatomic activities in response to these changes (Porges, 2007). Therefore, the vagal system is a factor in concurrent social, emotional, and hemodynamic regulation. Because of this, the patterns of social, emotional, and hemodynamic dysregulation observed in MDD patients could be related to dysregulation in their vagal control mechanisms (Cyranowski et al., 2011). The respiratory system affects the cardiac system mainly via vagal stimulation and direct mechanical impacts on the sinoatrial node (Yoon et al., 2018). The changes in blood and thoracic pressures related to respiratory movements, directly and indirectly, impact the sino-atrial node, resulting in fluctuations in heart rate and leading to heart rate variability (HRV) (Sola-Soler et al., 2018). Decreased heart rate variability (HRV) has been linked to depression and indicates a low level of cardiac control (Brown et al., 2018; Hartmann et al., 2019; Koch et al., 2019). Furthermore, OSA patients have more sympathetic activities than healthy individuals. Their HRV is substantially changed due to surges in heart rate associated with repetitive cycles of the apnea-hypopnea (Szollosi et al., 2007).

One of the Indirect indicators of cardiac vagal control is Respiratory Sinus Arrhythmia (RSA). RSA can be estimated from continuous ECG signals using spectral analyses. RSA is related to the high-frequency component (HF) of HRV and has been associated with the parasympathetic activity (Shader et al., 2018). Respiratory frequency and tidal volume have a big impact on RSA. The magnitude of RSA relies on the amplitude and frequency of the respiratory oscillations. If the respiratory frequency is kept constant, RSA will increase as the tidal volume increases (Cysarz and Büssing, 2005). RSA also can enhance pulmonary gas exchange to maintain high gas consumption efficiency (Giardino et al., 2003).

Recently, cardiorespiratory phase synchronization has gained more attention (Schäfer et al., 1998; Schäfer et al., 1999). Multiple studies have also investigated cardiorespiratory synchronization in OSA patients. Their analysis of sleep stages for OSA patients reflected a significantly higher cardiorespiratory synchronization in NREM sleep compared to REM sleep in OSA patients. This reduction of phase coupling in REM sleep was attributed to long-term associated noises from higher brain regions (Kabir et al., 2010; Sola-Soler et al., 2015). Investigators have also studied the phase coherence between the hemodynamic variable RSA and the respiratory system during induced mental stress. They showed how mental stress could affect the phase lag variations between RSA and respiratory. The study found that the degree of phase coherence was positively correlated with RSA amplitude and HF power, suggesting that the degree of phase coherence may also be associated with vagal activity. Additionally, they found that respiratory frequency decreased as phase coherence increased, but the mechanism was unclear (Niizeki and Saitoh, 2012).

These findings suggest that phase coherence might be an alternative measure for assessing the ANS. We assumed that if the stress can affect the phase coherence of RSA as a reflection of changes in the RSA amplitude and hence in the parasympathetic activities, depression caused by continuous cognitive stress could also impact the phase coherence of RSA. However, no study has ever investigated the phase coherence of RSA and respiration in depressed patients with OSA during nocturnal sleep. Thus, it remains to be answered if the phase coherence of RSA and respiration exists in OSA patients with and without MDD, and if it exists, how does it differ across the sleep stages? The present study aimed to investigate if the phase coherence of RSA and respiration can be used as a trait marker for distinguishing between depressed and non-depressed OSA patients.

## 2 Materials and methods

### 2.1 Participants and data collection

Overnight polysomnography was performed on 86 subjects at the American Center for Psychiatry and Neurology (ACPN) in Abu Dhabi, among which 40 had OSA with MDD (OSAD+), and 40 had only OSA without MDD (OSAD-), and 6 healthy subjects (controls). Data collection and use were approved by the Institutional Review Board (IRB) of the American Center for Psychiatry and Neurology in Abu Dhabi on the second of October 2017, with protocol number or IRB reference number (0019). All participants gave written consent. Exclusion criteria included: Patients having comorbid Cardiovascular diseases. All patients recruited in this study were diagnosed with OSA. The level of clinical depression was assessed by a consultant psychiatrist (VL) using the Patient Health Questionnaire-9 (PHQ-9) (Salkind, 1969). Only OSA patients who were identified with mental health issues completed the PHQ-9 questionnaire. The PHQ-9 is a 9-item self-reported questionnaire measuring the severity of depressive symptoms. For each item, the respondent chooses one or more response options rated from 0 (absence of symptom) to 3 (nearly every day). Total scores range from 0 to 27 and represent the sum of the highest level endorsed on each item. The PHQ-9 scores 5, 10, 15, and 20 represent mild, moderate, moderately severe, and severe depression, respectively (Kroenke et al., 2001). In this study, subjects with a positive score of 10 and above for PHQ-9 were categorized as having major depressive disorder (MDD). Additionally, 32 controls without OSA or major depressive disorder are included from the Stanford Technology Analytics and Genomics in Sleep (STAGES) dataset (Zhang et al., 2018). Same data were analyzed and used in the following research (Moussa et al., 2022).

ECG and piezoelectric belts based on thoracic movement signals were used in this study. They were recorded for ACPN data at sampling frequencies of 100 Hz and 10 Hz, respectively, and for STAGES data at a sampling rate of 200 Hz and 50 Hz, respectively.

### 2.2 Data analysis

**FIGURE 1:**
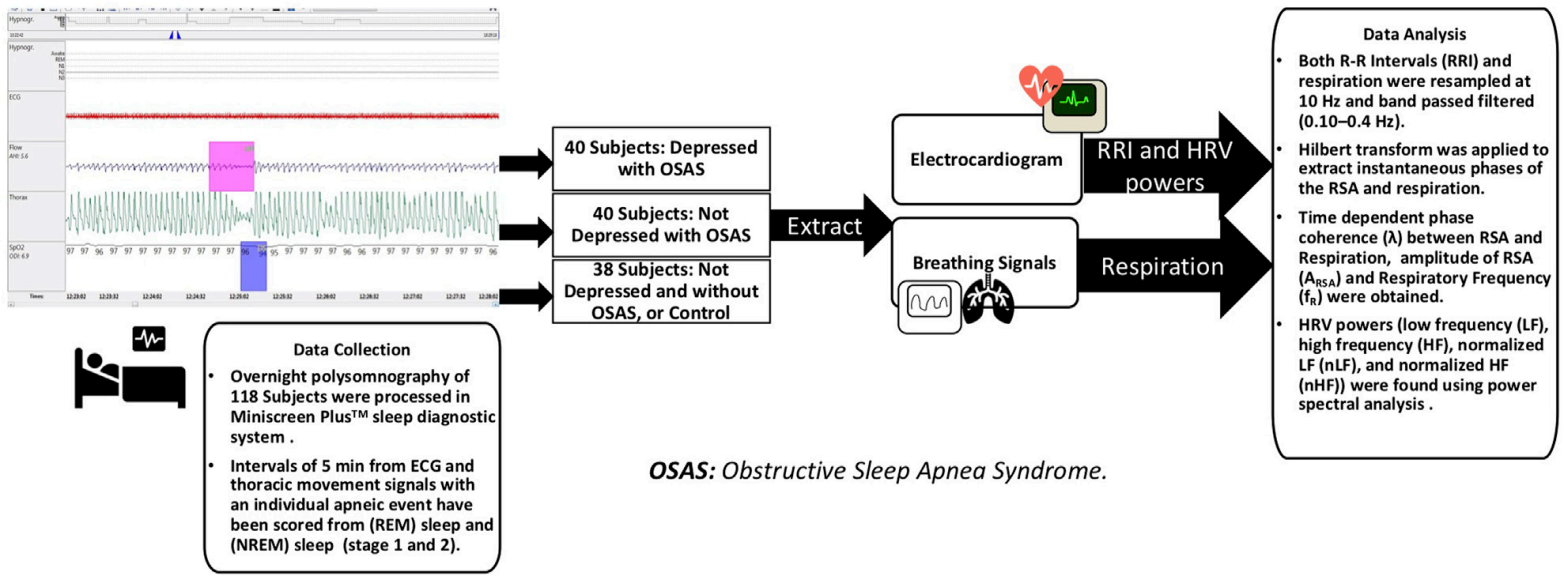
Data collection and analysis.

For the analysis, intervals of 5-min from ECG and thoracic movement signals with an individual apneic event have been manually and carefully selected from NREM sleep and REM sleep stages (stages 1 and 2). According to Task Force of the European Society of Cardiology and the North American Society of Pacing and Electrophysiology, 5-min segment is enough to obscure the detailed information about autonomic modulation of RR intervals that is responsible for autonomic indexes (Camm et al., 1996). On average, 3 intervals were selected from each sleep stage for each subject. For the patients with severe OSA, at least one segment was selected from each sleep stage due to difficulty of scoring 5-min intervals with a single OSA episode without the risk of scoring other respiratory event. 353 segments in NREM sleep and 298 segments in REM sleep were analyzed. Table 1 shows the distribution of the actual number of analyzed segments for OSAD+, OSAD-, and control groups during NREM and REM sleep. Apneic events in the intervals were scored as follows: An obstructive apnea is characterized by 10 s blockage of the oronasal airflow despite continuous chest and abdominal movements. An obstructive hypopnea is characterized by 10 s of partial blockage of the upper airway, causing a 50% decrease in the airflow (Schröder and O’Hara, 2005). No distinction between obstructive apnea and hypopnea was made when apneic events in the intervals were scored. To capture the entire effect before, during, and after the apneic event occurs on the selected segments. We chose apneic events to be preceded and followed by continuous breathing and start after a minimum of 1 minute and half from the beginning of the segment and end before a minimum of 1 minute and half from the ending of the segment. Figure 1 illustrates the data collection and analysis in this study.

**TABLE 1:** Number of analyzed segments for OSAD+, OSAD-, and control groups in both NREM and REM sleep stages.

	NREM	REM
OSAD+	127	98
OSAD-	131	119
Control	95	81
Total	353	298

The times of R-peak locations from the ECG signal were determined using the peak detection algorithm for R-wave. RRI was calculated as the time duration between two consecutive R peaks. The resulting RRI was visually inspected, and outliers were deleted. Then, the RRI was resampled at 10 Hz using a spline interpolation method. The respiratory movement was also resampled at 10 Hz. Both RRI and respiratory signals were high-pass-filtered at 0.1 Hz. Then low-pass filtered at 0.4 Hz using a finite impulse response (FIR) digital band-pass filter to remove variances. From the oscillatory signals of RRI and respiration obtained, instantaneous amplitude and phase were calculated using the Hilbert transform, which shifted phases of all frequency components of its input by a quarter cycle. The analytic signal Z(t) was found from a real value input, and its Hilbert transform was described by the following equation:

$$Z\left(t\right)=v\left(t\right)e^{-i\Phi \left(t\right)}$$

Where $v\left(t\right)$ and $\Phi \left(t\right)$ are instantaneous amplitude and phase of the analytic signal, respectively. The instantaneous phase $\Phi \left(t\right)$ shows phase jumps of ±2 π whenever it passes zero, therefore, the $\Phi \left(t\right)$ was unwrapped to a continuous function of time without distortions. The instantaneous phases at discrete k-th times t_k_ of the RRI [ Φ_RRI_ (t_k_)] and respiration[Φ_RESP_ (t_k_)] were used to calculate the time dependent phase coherence (λ) which assessed the strength of phase locking between RSA and Respiration. To calculate λ, the phase difference ¥(t_k_) = [ Φ_RRI_ (t_k_) - Φ_RESP_ (t_k_)] modulo 2 π was defined and then the following equation was used:

$$\lambda \left(\text{tk}\right)={\left|\frac{1}{N}\sum_{j=k-N}^{k}e^{i¥\left(\text{tk}\right)j}\right|}^{2}$$

Where N denotes the number of data samples and λ always ≤1. The λ values were calculated from 300-point windows (30 s) with a 50-point sliding window. The amplitude of RSA (A_RSA_) was calculated using the average instantaneous amplitude of oscillatory signal of RRI ( $\left.v\left(t\right)\right)$ . The Respiratory Frequency (f_R_) was calculated by dividing the Φ RESP(t) derivative by 2 π as a function of time (Niizeki and Saitoh, 2012; Khandoker et al., 2018).

**FIGURE 2:**
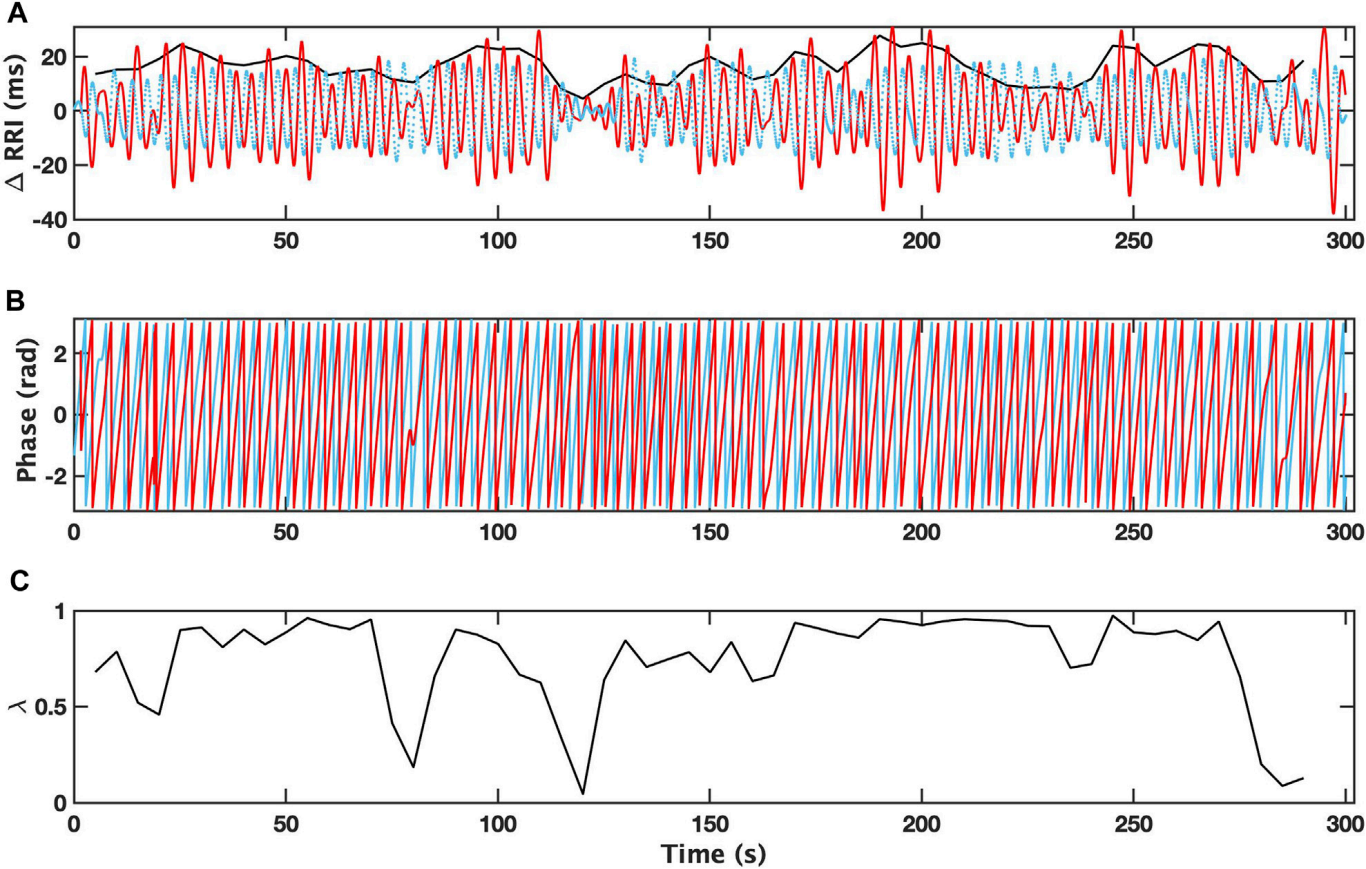
Changes in R-R intervals (RRI; solid red curve) and breathing trace (in the arbitrary unit; dotted blue curve) for 5 min-interval with a single apneic event from a patient with depression (OSAD+) during NREM are shown **(A)**. Bold black curves indicate the instantaneous amplitude of RRI. Instantaneous phases for RRI (Φ_RRI_ (t), red solid curve) and breathing (Φ_RESP_(t), blue solid curve) are shown **(B)**. The time dependent phase coherence (λ) produced from this 5 min-interval is also shown **(C)**.

An example of the analysis for a 5-min interval with a single apneic event from patient with depression (OSAD+) during NREM is demonstrated in Figure 2, where the filtered RRI (solid red curve) and simultaneous respiratory pattern (dotted blue curve) for OSAD+ (Figure 2A) are shown. The Hilbert transform of these signals gives the instantaneous phase of Φ_RRI_ (t) (red solid curve) and Φ_RESP_(t) (blue solid curve) (Figure 2B), and the amplitude $v\left(t\right)$ [bold black curves in (Figure 2A)]. RRI(t) and RESP(t) exhibited a saw tooth pattern, with RESP(t) slightly leading RRI(t). The time-dependent phase coherence (λ) produced from this 5-min interval is shown in (Figure 2C), λ values approach unity when the phase locking between the two oscillations is stronger.

#### 2.2.1 Heart rate variability (HRV)

To assess autonomic activities, components of HRV were computed. Spectral analysis using Welch’s method was performed on a linearly resampled (4 Hz) time series (Welch, 1967). The 256-point fast Fourier transform was repeatedly computed with a fifty per cent overlap between adjacent segments. Then, the spectral power of each segment was computed and averaged. Hanning window was used to avoid spectral leakage. Subsequently, the low-frequency band (LF; 0.04–0.15 Hz) and high-frequency (HF) band (HF; 0.15–0.40 Hz) were obtained by integration (Camm et al., 1996). Normalized index of HF and LF powers were also computed by HF/(LF + HF) and LF/(LF + HF), respectively (Burr, 2007).

### 2.3 Statistical analysis

Nonparametric Mann-Whitney *U*-test was used to check the differences between OSA patients with MDD (OSAD+), OSA patients without MDD (OSAD-), and healthy control subjects during both REM and NREM sleep stages. The correlations between λ and autonomic variables (A_RSA_, HF, LF, nHF and nLF), including f_R_ were evaluated by the linear least squares regression analysis. Values of *p* < 0.05 were considered statistically significant for all comparisons.

## 3 Results

Demographic characteristics and AHI values of all subjects are displayed in Table 2. It is also noted that Table 2 indicates no significant difference in terms of age and BMI across the three groups of subjects, which can eliminate ageing and weight-driven biases. In addition, Table 2 showed no significant difference in terms of AHI for both NREM and REM sleep between OSA patients with and without depression, which can eliminate OSA severity impact on the results.

**TABLE 2:** Demographic data and AHI values of participants’ findings in different groups of OSA patients and control group.

	OSAD+	OSAD-	Control	Probabilities (MAN-Whitney U-test for groups)		
				OSAD + vs. OSAD-	OSAD- vs. control	OSAD + vs. control
Participants	(40)	(40)	(38)	—	—	—
Gender(M/F)	(21/19)	(27/13)	(12/26)	—	—	—
Age	43 ± 10	45 ± 11	40 ± 14	ns	ns	ns
BMI (kg/m^2^)	31 ± 6	33 ± 10	30 ± 6	ns	ns	ns
PHQ-9	16.8	—	—	—	—	—
(AHI/hr)	23 ± 15	23 ± 15	1.7 ± 1.5	ns	ns	<0.001
NREM (AHI/hr)	29 ± 20	24 ± 18	1.8 ± 1.8	ns	<0.001	<0.001
REM (AHI/hr)	31 ± 24	29 ± 22	2.1 ± 3.3	ns	<0.001	<0.001

*ns indicates no significant difference.

Table 3 summarizes group comparisons for λ, f_R_ **,** and autonomic variables (A_RSA_, HRV and normalized HRV index) during NREM and REM sleep. In NERM sleep, higher λ was found in the OSAD + group compared to both OSAD- and Control groups while lower λ was found in OSAD-group compared to Control group. The Control group had significantly higher f_R_ compared to both OSAD+ and OSAD-groups. The Control group also had significantly lower A_RSA_ compared to OSAD-groups. In REM sleep, higher λ was found in the OSAD + group compared to the OSAD-group and lower λ was found in the OSAD + group compared to the Control group. In addition, λ was significantly lower in OSAD-group compared to Control group. Similar to NREM sleep, the Control group had significantly higher f_R_ compared to both OSAD + group and OSAD-group. The Control group also had significantly lower A_RSA_ compared to both OSAD+ and OSAD-groups. Neither f_R_ nor A_RSA_ had a significant difference between OSAD+ and OSAD-groups during both sleep stages.

**TABLE 3:** Effects of OSA and depression on cardiorespiratory and autonomic variables during NREM and REM sleep.

	OSAD+	OSAD-	Control	Probabilities (MAN-Whitney U-test for groups)		
				OSAD + vs. OSAD-	OSAD- vs. control	OSAD + vs. Control
NREM Sleep						
f_R_(breaths/min)	16.6 ± 2.4	17.3 ± 3.0	21.4 ± 3.6	ns	<0.001	<0.001
A_RSA_(ms)	26.6 ± 40.3	34.1 ± 39.8	19.1 ± 13.0	ns	0.018	ns
λ	0.67 ± 0.13	0.56 ± 0.19	0.63 ± 0.12	<0.001	0.006	0.02
LF (bpm^2^)	3.54 ± 4.22	4.61 ± 4.61	5.53 ± 7.20	0.03	ns	ns
HF (bpm^2^)	1.56 ± 1.55	2.08 ± 3.18	2.52 ± 4.51	ns	ns	ns
nLF	65.4 ± 18.8	70.8 ± 19.0	69.8 ± 18.3	0.013	ns	ns
nHF	34.6 ± 13.6	29.3 ± 19.0	30.3 ± 18.3	0.013	ns	ns
REM Sleep						
f_R_(breaths/min)	17.2 ± 2.1	17.4 ± 2.8	22.0 ± 3.1	ns	<0.001	<0.001
A_RSA_(ms)	31.2 ± 45.0	26.9 ± 37.4	18.7 ± 22.8	ns	0.028	0.002
λ	0.57 ± 0.15	0.50 ± 0.17	0.62 ± 0.12	0.006	<0.001	0.02
LF (bpm^2^)	3.81 ± 3.79	5.08 ± 4.75	4.64 ± 5.91	0.04	ns	ns
HF (bpm^2^)	1.43 ± 1.62	1.70 ± 2.83	2.05 ± 3.92	ns	ns	ns
nLF	71.9 ± 15.9	76.9 ± 16.5	75.1 ± 15.2	0.009	ns	ns
nHF	28.2 ± 15.9	23.1 ± 16.4	24.9 ± 15.2	0.009	ns	ns

*ns indicates no significant difference.

Table 3 also shows which HRV parameters separate between the three groups during both NREM and REM sleep stages. In NREM sleep, LF power and normalized LF were significantly decreased, while normalized HF was significantly increased in the OSAD + group compared to OSAD-group. In the REM sleep stage, LF power and normalized LF were significantly decreased, while normalized HF was significantly increased in the OSAD + group compared to OSAD-group. No significant difference was found in HF between the three groups in both sleep stages.

**FIGURE 3:**
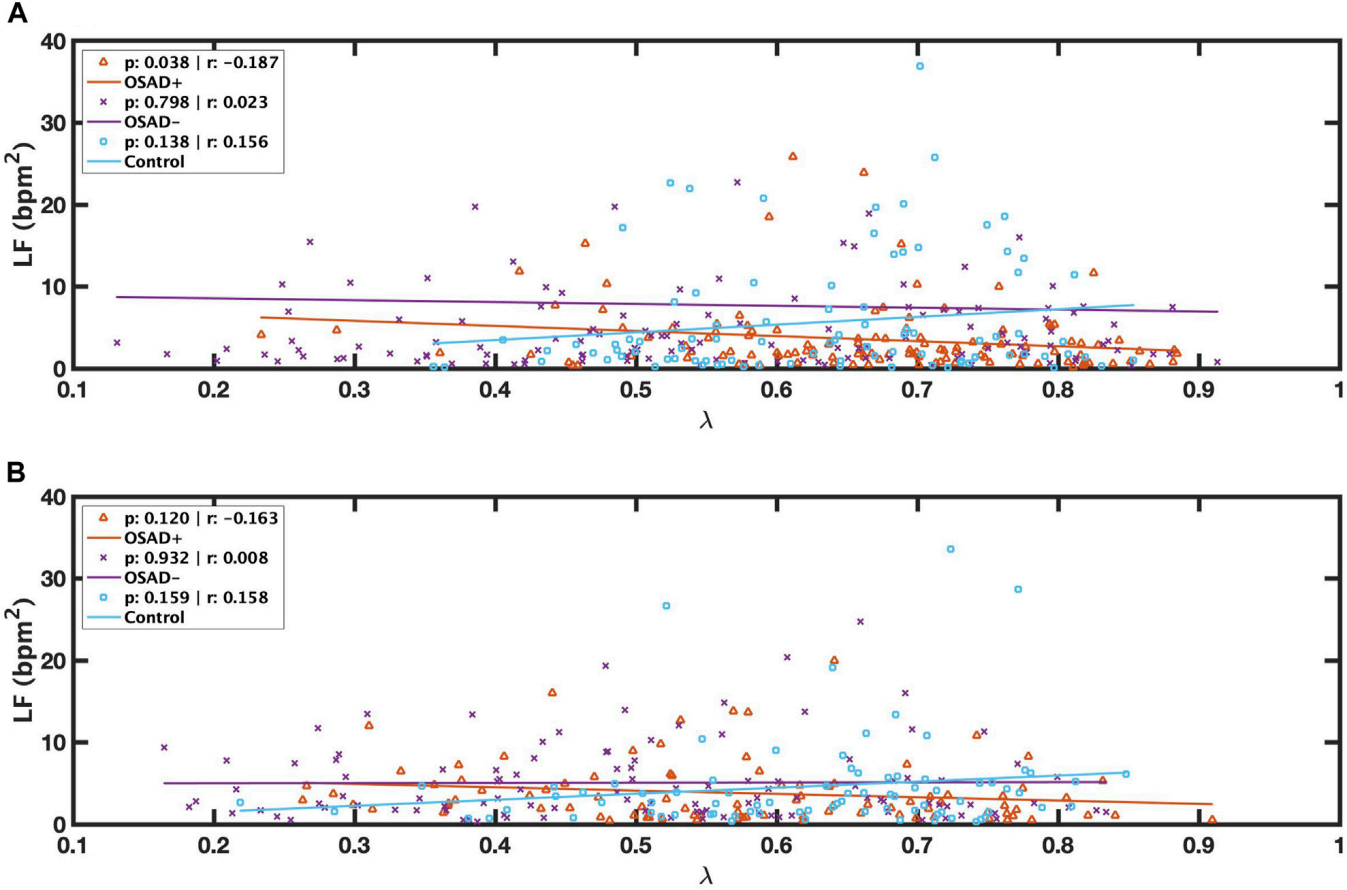
Scatter plots of relations between λ and LF during NREM **(A)** and between λ and LF during REM **(B)** for OSAD+ (solid orange line), OSAD- (solid purple line), and Control (solid blue line) groups.

Bivariate correlations among λ, f_R_, A_RSA,_ HRV, and normalized HRV index in NREM and REM sleep are shown in Table 4. The λ was inversely related to LF (r = −0.187; *p* = 0.038) in the OSAD + group only in NREM sleep and was positively associated with HF in OSAD-group in the both NREM (r = 0.363; *p* = 0.000) and REM (r = 0.195; *p* = 0.043) sleep stages, whereas no correlation was observed between λ and A_RSA_ for the three groups during both sleep stages. Negative correlation coefficients with (*p* < 0.01) were found between λ and normalized LF in both OSAD+ and OSAD-for both NREM and REM sleep stages, whereas positive correlation coefficients with (*p* < 0.01) were found between λ and normalized HF in both OSAD+ and OSAD-for both sleep stages as well. For the Control group, λ was inversely related to nLF (*r* = −0.338; *p* = 0.002), and positively correlated to nHF (*r* = 0.338; *p* = 0.002) only in the REM sleep. Moreover, Figures 3A, B also show that λ was negatively associated with LF only in OSAD + group during NREM sleep.

**TABLE 4:** Bivariate correlations among λ, f_R_, A_RSA_, HRV, and normalized HRV index in NREM and REM sleep for OSAD+, OSAD- and Control groups.

	OSAD+		OSAD-		Control		OSAD+		OSAD-		Control	
			NREM sleep						REM sleep			
	*p*	*r*	*p*	r	*p*	*r*	*p*	*r*	*p*	*r*	*p*	*r*
f_R_ (breaths/min)	**0.001**	**−0.289**	**0**	**−0.571**	**0**	**0.432**	**0**	**−0.481**	**0**	**−0.538**	**0**	**0.703**
A_RSA_ (ms)	0.733	0.0311	0.575	−0.0504	0.903	−0.013	0.1332	0.0714	0.164	0.1342	0.986	0.002
LF (bpm^2^)	**0.0381**	**−0.187**	0.798	0.0231	0.138	0.156	0.112	−0.163	0.9325	0.0082	0.160	0.158
HF (bpm^2^)	0.0685	0.165	**0**	**0.362**	0.605	0.0547	0.0566	0.1994	**0.0426**	**0.195**	0.232	0.134
nLF	**0**	**−0.380**	**0**	**−0.640**	0.931	0.0091	**0**	**−0.602**	**0**	**−0.453**	**0.002**	**−0.338**
nHF	**0**	**0.380**	**0**	**0.640**	0.931	−0.009	**0**	**0.602**	**0**	**0.453**	**0.002**	**0.338**

Bold value indicates the significant correlation.

**FIGURE 4:**
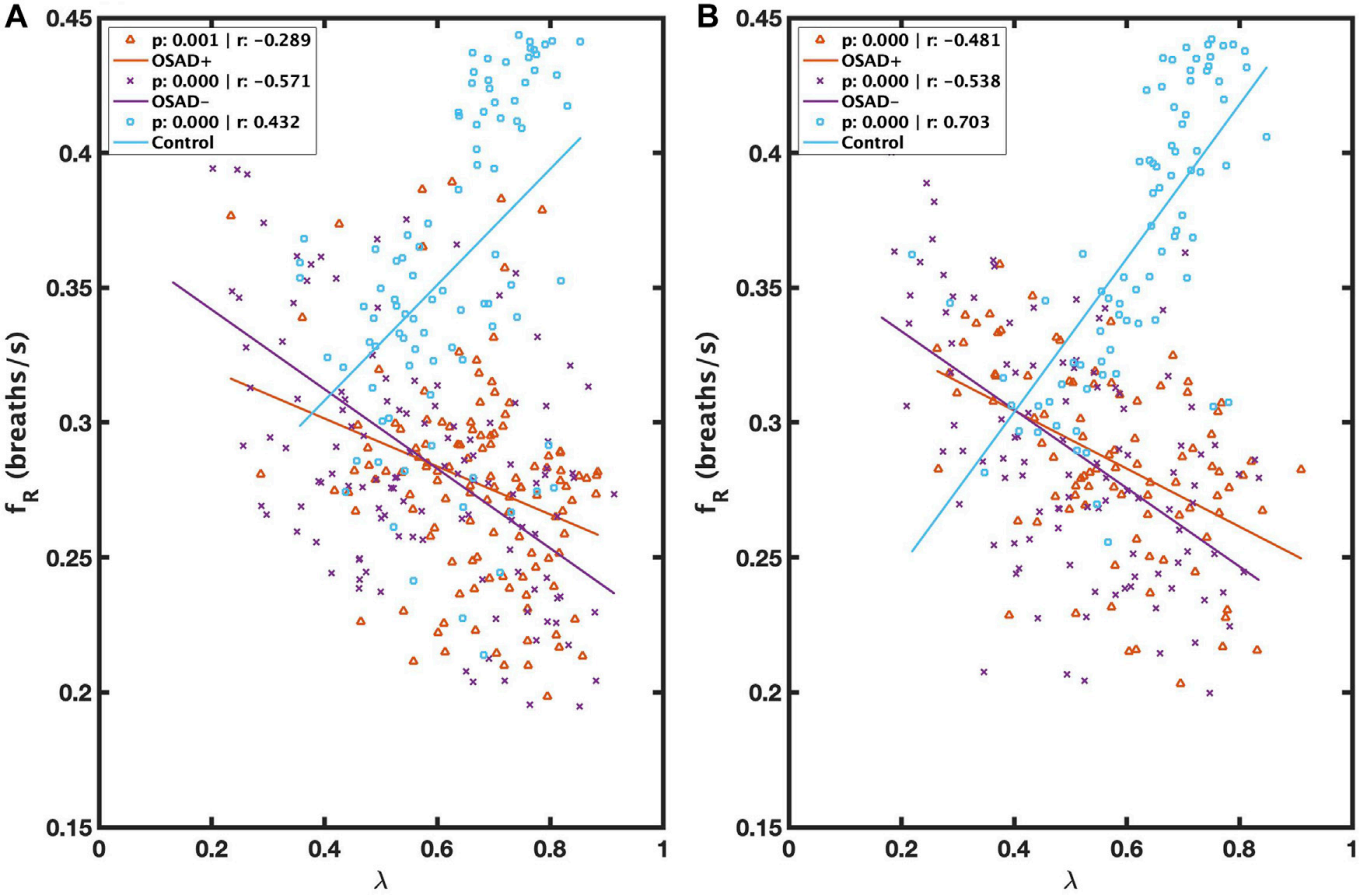
Scatter plots of relations between λ and f_R_ during NREM **(A)** and between λ and f_R_ during REM **(B)** for OSAD+ (solid orange line), OSAD- (solid purple line), and Control (solid blue line) groups.

Figure 4 illustrates the correlation between λ and f_R_ during NREM and REM sleep. λ was inversely related to f_R_ in both OSAD+ (solid orange line) and OSAD- (solid purple line) groups for NREM (Figure 4A) and REM (Figure 4B) sleep, while λ was positively correlated to f_R_ in the Control group (solid blue line) for NREM (Figure 4A) and REM (Figure 4B) sleep.

**FIGURE 5:**
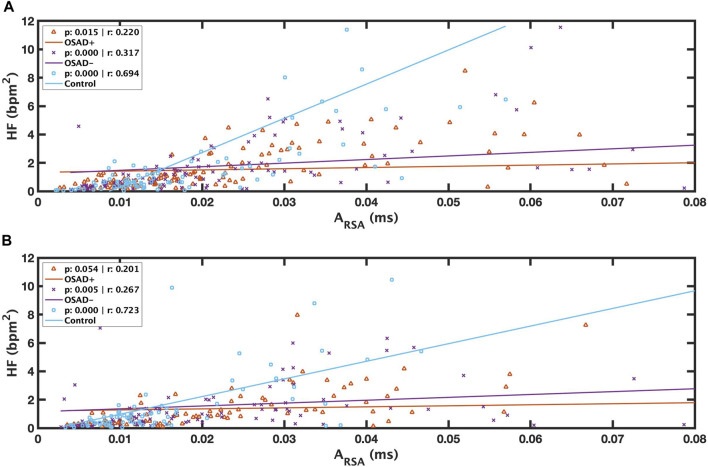
Scatter plots of relations between λ and A_RSA_ during REM **(A)** and between λ and A_RSA_ during NREM **(B)** for OSAD+ (solid orange line), OSAD- (solid purple line), and Control (solid blue line) groups.

Relationships among A_RSA_, f_R_, HRV, and normalized HRV index in NREM and REM sleep are shown in Table 5. Positive correlation coefficients were found between A_RSA_ and HF. Figure 5 presents these correlations between RSA and HF during NREM and REM sleep. For the Control group, a high correlation coefficient was found between A_RSA_ and HF in both NREM (solid blue line in Figure 5A) and REM (solid blue line in Figure 5B) sleep stages. The OSAD+ (solid orange line) and OSAD- (solid purple line) groups also showed significant positive correlations between A_RSA_ and HF almost everywhere except that the OSAD + group in REM (Figure 5B) sleep did not reach statistical significance with (*r* = 0.201 and *p* = 0.054).

**TABLE 5:** Bivariate correlations among A_RSA_, f_R_, HRV, and normalized HRV index in NREM and REM sleep for OSAD+, OSAD- and Control groups.

	OSAD+		OSAD-		Control		OSAD+		OSAD-		Control	
			NREM sleep						REM sleep			
	*p*	*r*	*p*	*r*	*p*	*r*	*p*	*r*	*p*	*r*	*p*	*r*
f_R_ (breaths/min)	0.803	0.0247	**0.001**	**−0.292**	0.3531	0.0943	0.742	0.0347	**0.0351**	**−0.202**	0.680	−0.0466
LF (bpm^2^)	**0.0388**	**0.203**	0.0904	0.151	**0.0018**	**0.309**	0.134	0.158	0.0866	0.165	**0.0001**	**0.433**
HF (bpm^2^)	**0.015**	**0.220**	**0.0003**	**0.317**	**0**	**0.676**	0.0543	0.201	**0.005**	**0.267**	**0**	**0.723**
nLF	0.411	0.0815	0.131	−0.135	**0**	**−0.430**	0.975	−0.003	0.131	−0.146	**0.0003**	**−0.390**
nHF	0.411	−0.0815	0.131	0.135	**0**	**0.632**	0.975	0.003	0.131	0.146	**0.0003**	**0.390**

Bold value indicates the significant correlation.

**FIGURE 6:**
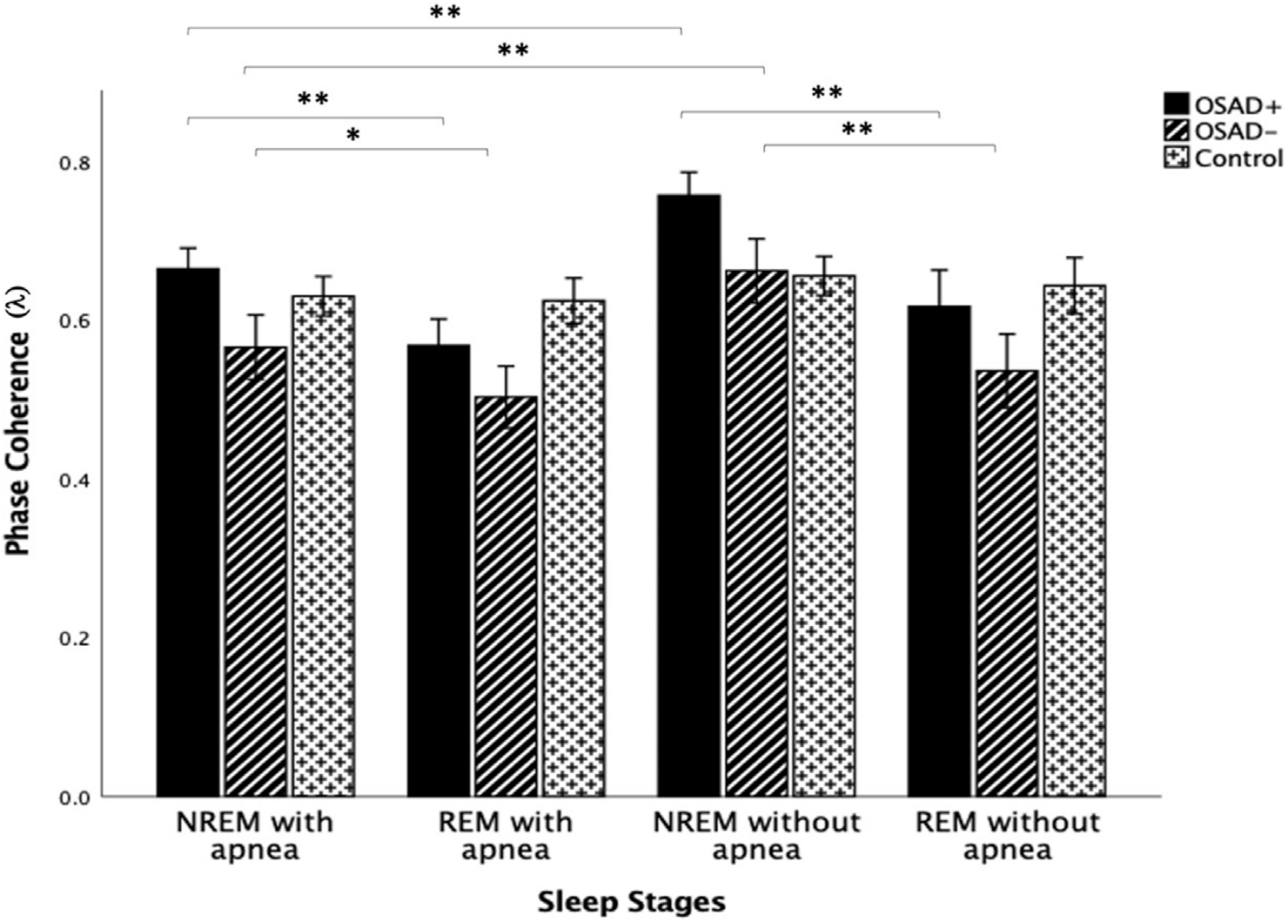
Group and sleep-related comparison for λ including the significance levels of difference according to the existence of the apneic event. * indicates the significant differences *p* < 0.05 and ** indicates the significant differences *p* < 0.001.

Figure 6 depicts group comparisons for λ, including the significance levels of difference according to the different sleep stages and the existence of apneic events. λ significantly increased in OSAD+ and OSAD-groups during the NREM sleep stage compared to the REM sleep stage in both cases with and without apneic event. At the same time, no significant difference in λ was observed between the sleep stages in the control group. Furthermore, λ significantly decreased in OSAD+ and OSAD-groups during NRME sleep with apnea compared to NREM sleep without apnea. There was also a decrease in λ values in OSAD+ and OSAD-groups during REM sleep with apnea compared to the REM sleep without apnea; however, they did not reach statistically significant levels. No significant difference between sleep stages with and without apnea was observed for the control group.

## 4 Discussions

The present study demonstrated that depression increased synchronization between RSA and respiration, assessed by the phase coherence (λ) among OSA patients during nocturnal light NREM and REM sleep stages. These variations in λ could not be ascribed to variations in RSA or f_R,_ as no significant difference was found between OSAD+ and OSAD-groups in these variables. In addition, λ did not show a significant correlation to RSA, and only a negative relationship was observed between λ and f_R_ for both groups. However, it was found that the increase in λ was associated with a decrease in the LF component of HRV and its normalized index nLF, suggesting a possibility that depression might have lowered the sympathetic activity among patients with OSA, allowing for more robust synchronization between RSA and respiration. This section is divided into six sub-sections to improve readability, followed by a limitation section.

### 4.1 Effect of depression on phase coherence (λ) in OSA patients

This study aimed to investigate if there is any difference in λ in OSA patients with and without MDD. λ was significantly increased in the OSAD + group compared to OSAD-group during NREM and REM sleep (Table 3). This might be due to a reduction in sympathetic cardiac activities, making the RSA rhythm less erratic, and therefore phase-locking with respiratory signals is more likely to occur. Several investigators have demonstrated that depression decreases the HRV, including the LF power in humans (Brown et al., 2018; Hartmann et al., 2019; Koch et al., 2019). In this study, LF power significantly decreased in the OSAD + group during NREM and REM sleep (Table 3). The change in LF power can be due to sympathetic and parasympathetic activities in ANS. Still, since the normalized index nLF had significantly decreased (Table 3), the decrease in LF could be attributed to the reduction of the sympathetic activity (Billman, 2013).

The precise mechanism(s) responsible for the effect of the sympathetic activity on the phase coherence (λ) is unclear. A possible explanation is that the sympathetic nerve activity may alter the transduction property of the cardiac vagal efferent nerve (Taylor et al., 2001). There is evidence that neuromodulators called neuropeptide Y are co-released with norepinephrine from sympathetic nerve terminals, thereby exerting an inhibitory action on the vagally mediated heart periods oscillations (Potter, 1985), suggesting that the high levels of sympathetic activity may decrease the magnitude of RSA, which in turn can affect the degree of phase coherence (λ). Given that sympathetic activity be reduced by depression, an increase in the degree of phase coupling of RSA in the OSAD + group compared to OSAD may indicate the predominance of vagal activity in the depressed OSA group. Perhaps related to the effect of neuropeptide Y on parasympathetic effect or junctions of the heart, in less sympathetic stimulation, less neuropeptide Y would be released from sympathetic nerve terminals and thereby, less inhibition would be excreted on the vagal innervation of the heart. The predominance of vagal activity can also be explained by the significant increase in the normalized index of HF in the OSAD + group compared to the OSAD-group.

### 4.2 Effect of OSA on respiratory frequency (f_R_) and amplitude of RSA (A_RSA_)

It was found that the OSAD+ and OSAD-groups had significantly lower f_R_ and higher RSA amplitude than the control group during NREM and REM sleep (Table 3). This result agrees with a previous study in which f_R_ and amplitude of RSA were found to be reciprocal (Cysarz and Büssing, 2005). In this study, although we used only 5-min intervals including an individual apneic event for the analysis, we still found that the respiratory rate substantially differed between the two groups. An explanation for this observation of a lower respiratory frequency in OSA patients might be due to impairment in the signalling pathway of the respiratory stimulant adipose-derived hormone leptin. It is reported that OSA syndrome can lead to higher circulating leptin levels. These high leptin levels are related to leptin-resistant and impaired leptin singling in the brain, causing respiratory depression (Berger and Polotsky, 2018).

Furthermore, the higher amplitude of RSA observed in OSA^+/−^patients can be due to stimulation of the carbon dioxide chemoreceptors. When atrial CO_2_ is elevated during the apnea episode, RSA amplitudes increase to evacuate excess levels of CO_2_ in the pulmonary circulation and enhance the pulmonary gas exchange (Hayano and Yasuma, 2003). This might explain the augmentation of RSA amplitude in OSA patients.

### 4.3 Effect of OSA on phase coherence (λ)

λ was significantly decreased in OSAD-group compared to the control group in light NREM and REM sleep (Table 3). Because of the significant differences in f_R_ between the two groups, the differences in RSA cannot be interpreted as a difference in cardiac vagal tone between the two groups. Several studies reported that whenever respiratory parameters substantially differ between the groups, employing RSA solely as an index of cardiac tone should be interpreted with caution (Grossman et al., 1991; Grossman and Kollai, 1993; Cyranowski et al., 2011). Thus, the significant difference in λ between the two groups cannot be discernibly tracked by the attenuations in RSA magnitude under variable respiratory rates. Our results confirmed this; no correlation was found between λ and RSA (Table 4). A possible explanation for the low values of λ in the OSAD-group compared to the control group is that OSA subjects have been reported to have higher sympathetic activity, mainly due to heart rate fluctuations before and after the apneas and sympathetic arousals at the end of apneic events (Narkiewicz and Somers, 2003). Despite using 5-min intervals with single apneic episodes for the analysis in both control and OSAD-groups to minimize the differences between both groups, we would still expect the sympathetic tone to be higher in OSAD-patients than in control subjects. This might be due to dysfunction in the baroreflex (Narkiewicz et al., 1998a) and chemoreflex (Narkiewicz et al., 1998b) caused by repeated episodes of hypoxia and cyclical increases in arterial pressure, which leads to elevated blood pressure and chronic activation of the sympathetic nervous system. A persistent enhancement in sympathetic tones could lead to cardiac electrical remodeling, thus facilitating rhythm dysfunction, particularly atrial fibrillation (Allessie, 2002). It has been reported that patients with moderate to severe OSA exhibited expansion of the left ventricular mass and dysfunctional diastolic pressure, common reasons for high blood pressure (Fung et al., 2002), but not those with mild OSA (Niroumand et al., 2001), suggesting that moderate to severe OSA patients would have chronic sympathetic outflow compared to mild OSA patients and healthy individuals. Other intriguing possibilities are that such lower values of λ in OSAD-patients compared to healthy subjects might be related to differences in both airway anatomies and upper airway muscle resistance in OSA patients. Most OSA patients exhibit growth of the soft tissue structures within and surrounding the airway, which might contribute substantially to pharyngeal airway narrowing (Ciscar et al., 2001). In addition, OSA patients have less upper airway resistance (Dempsey et al., 2010). Because of these factors, we would expect arousals after apnea episodes to be higher and values of λ to be lower in the OSAD-group compared to the control group.

Mixed results were found for λ values in the OSAD + group compared to the control group (Table 3). λ values varied with sleep stages. λ was significantly higher during NREM sleep in the OSAD + group compared to the control group. In addition, LF was inversely related to λ in NREM sleep (Table 4) (Figure 3A), suggesting that sympathetic activity has decreased during NREM for depressed OSA patients compared to healthy individuals, which might explain the significant increase in the phase locking between RSA and respiration. Despite OSA patients having higher sympathetic activities compared to healthy individuals (Szollosi et al., 2007; Kabir et al., 2010), our results, in contrast, showed that sympathetic activity was decreased in OSAD + group. This might be due to the impact of depression in reducing HRV.

On the other hand, it was found that λ was significantly lower during REM sleep in the OSAD + group compared to the control group. Similar findings were encountered when we reached the OSAD-group of healthy subjects (Table 3). An explanation for this observation of a lower λ during REM sleep is that sympathetic tone is dominant in the REM sleep stage compared to NREM sleep (Penzel et al., 2003; Sola-Soler et al., 2015), and therefore the decrease in sympathetic activity caused by depression may be negligible. This could also be why no significant difference in LF was observed during the REM sleep stage between the OSAD+ and the control groups (Table 3). Also, this might be why the correlation between λ and LF was lost (Table 4) (Figure 3B).

### 4.4 Relationship of phase coherence (λ) with respiratory frequency (f_R_)

λ was negatively related to f_R_ in both OSAD+ and OSAD-groups for NREM and REM sleep (Figures 4A, B). These observations might be because of the effect of respiratory rate on the amplitude of RSA. Several investigators who studied phase coherence between RSA and respiratory movements reported that the RSA amplitude under controlled respiratory parameters is negatively related to the respiratory frequency and directly related to the phase coherence (λ) (Cysarz and Büssing, 2005; Niizeki and Saitoh, 2012), implying that respiratory frequency and λ are inversely related. In contrast, it was observed that λ was positively correlated to f_R_ in control subjects for NREM and REM sleep (Figures 4A, B). A possible explanation for this observation is that in a healthy human, any obstruction on the upper airway muscle tonic activity during sleep will lead to a doubling, or even sometimes, a quadrupling resistance of the upper airway and swings of the intrathoracic pressure, which can cause a high breathing rate. As a result, only minor effects will be observed in the pulmonary gas exchange and autonomic regulations (Dempsey et al., 2010). The control subjects we used in this study had an AHI below five; therefore, the signals can still detect OSA episodes. To ensure the analysis is unbiased, we selected 5-min segments with a single apneic event from the control subjects. Thus, an increase in respiratory frequency was observed in these segments to minimize the apneic effects on the pulmonary gas exchange and the central nervous system. Since the pulmonary gas exchange and the central nervous system were more stable in these segments, the synchronization between RSA and respiration movements was stronger. This might explain the positive correlation between λ and respiratory frequency for healthy subjects.

### 4.5 Relations between HF and RSA among the groups

Our findings indicated that the changes in cardiac vagal tone assessed by HF power were tracked by changes in RSA amplitude in the same group with similar conditions (Figures 5A, B). These results agree with the literature. Several studies have reported positive correlations between RSA and HF power and between λ and HF power (Cysarz et al., 2004; Cysarz and Büssing, 2005; Niizeki and Saitoh, 2012). Our results also showed a positive correlation between λ and HF power only in the OSA patients in both NREM and REM sleep (Table 4). However, there was no relation between λ and HF power among depressed OSA patients or the control group. This could be because the depression might have altered the HRV for the OSAD + group, and the high breathing rate might affect the autonomic regulations in the control group.

### 4.6 Effects of sleep stages and existence of apneic event on phase coherence (λ)

λ significantly increased in both OSAD+ and OSAD-groups during NREM sleep compared to REM sleep in both cases with and without an apneic event (Figure 6). Multiple studies also investigated cardiorespiratory synchronization in OSA patients (Bartsch et al., 2007; Sola-Soler et al., 2015; Sola-Soler et al., 2016). However, the synchronization between heartbeats and the onset of respiratory cycle was used in these studies, which differs from the interaction between RSA and respiration. This interaction between heartbeats and respiratory signals occurs due to phase locking between the two oscillations, as heartbeats are generated at similar relative phases within consecutive respiratory cycles. Their analysis of sleep stages for OSA patients reflected a significantly higher cardiorespiratory synchronization in NREM sleep compared to REM sleep in OSA patients. This reduction of phase coherence in REM sleep could be due to long-term associated noise from higher brain regions. The higher brain region is more active during REM sleep, producing more noises that disturb the occurrences of the phase coupling (Sola-Soler et al., 2015). In addition, it was found that RSA decreases in REM sleep and dominates in NREM sleep. RSA can enhance pulmonary gas exchange, which may be associated with a noticeably reduced gas exchange during REM sleep compared to that during NREM sleep (Yoon et al., 2018). Again, considering the RSA as a major part of heartbeat fluctuation, their observations of an increase in the degree of phase synchronization between heartbeat and respiration during NREM sleep is comparable to our observation that the phase coupling of RSA with respiratory movements increased during NREM sleep compared to REM sleep in OSA patients. Moreover, the exitance of depressive symptoms in OSA patients did not change the fact that the phase coherence λ dominates in NREM over REM sleep.

Our results also showed that λ significantly decreased in both OSAD+ and OSAD-groups during NRME sleep with apnea compared to NREM sleep without apnea; however, the decrease in λ values was not significant during REM sleep with apnea compared to REM sleep without apnea (Figure 6). A possible explanation for these observations is that REM sleep is characterized with a fast and highly variable respiratory frequency like wakefulness. In contrast, NREM sleep is known as slow wave sleep (Dempsey et al., 2010). Thus, in an apneic event, we will observe only a little destruction to the respiratory rhythm in REM sleep. In contrast, in NREM sleep, the apnea will cause significant destruction to the respiratory rhythm. As a result, there will be a substantial change in autonomic regulations in NREM sleep. Based on that, we expect λ values to be significantly less in NREM sleep with apnea than in NREM sleep without apnea.

Furthermore, our results also showed that no significant change was found for the control group in λ values between NREM and REM sleep (Figure 6). This is attributed to the fact that the five minute-intervals that we used for the analysis in the control group exhibited a very high respiratory rate with very close values in both NREM and REM sleep (Table 3). Since respiratory rates significantly influence the RSA amplitude Field (Taylor et al., 2001), we expect RSA amplitude to be similar in both sleep stages and, consequently, the degree of phase coherence (λ). Therefore, λ values did not differentiate between NREM and REM sleep. Additionally, no variation in λ values was observed between the cases with and without apnea in the control group (Figure 6). This might be due to the high patency and resistance of healthy subjects’ upper airways, which do not allow apnea effects to be observed in the phase coherence (λ).

## 5 Limitations and future directions

While the results of this study provide numerous insights, a few limitations are noted. First, isolating 5-min intervals with a single OSA episode is easier for severe cases of OSA with the risk of scoring other hidden apneic events. Although we carefully and manually selected the 5-min intervals in this study, scoring such intervals for the severe cases of OSA might be a limitation. In this study, we had chosen all our segments with only one OSA episode to ensure that the OSA effect on RSA and respiration was consistent in each segment. For instance, the phase lag between RSA and respiration in segments with more than one OSA episode would have been more disturbed than in segments with only one OSA episode. Therefore, to ensure the analysis was unbiased, we ensured that all the segments in this study had an individual OSA event only. Second, we did not consider the respiratory rate variability (F_R_V) in this study. An earlier study showed that F_R_V could affect RSA and, in turn, could affect λ, as F_R_V has a short-term effect on the autonomic nervous system (Soni and Muniyandi, 2019). Also, F_R_V varies according to sleep stages, being greater in wakefulness than others in sleep stages (Gutierrez et al., 2016). Hence, future research can benefit from investigating the correlations among F_R_V, RSA and λ across sleep stages. Third, we did not measure tidal volume in this study. Tidal volume has a significant influence on RSA outcomes. For instance, increases in tidal volume at slower respiration rates will cause more significant RSA elevations than the same tidal volume growth at more rapid respiration rates (Cyranowski et al., 2011).

Moreover, apnea episodes cause a steady increase in the tidal volume, which could also affect RSA outcomes (Dempsey et al., 2004). The effect of tidal volume on λ for this study remains to be shown. However, this study investigated whether phase coherence (λ) could differentiate between OSA patients with and without depression. Recently, λ has been proposed as an alternative measure for evaluating autonomic nervous system (ANS) activity and subsequential as a measure of stress level (Niizeki and Saitoh, 2012). In this work, we have used λ to evaluate depression in OSA patients during nocturnal sleep. The changes in the λ between the groups were related to changes in the HRV index. Additionally, the heart rate varies along the night due to a circadian rhythm of HRV(Da Silva et al., 2009; Boudreau et al., 2011). This may also have effect on λ. Future research should investigate the circadian effect on the degree of phase coherence between RSA and respiration in OSA patients with and without MDD and how this degree of phase coherence (λ) may change by sleep cycles.

## 6 Conclusion

In summary, we examined the cardiorespiratory phase coherence during nocturnal sleep in OSA patients with and without depression, as well as healthy individuals. We found that depression could lower the sympathetic activity when accompanied by OSA, allowing for more robust synchronisation between RSA and respiration. We also found that change in the λ values between OSA patients with and without MDD could not be ascribed to change in RSA or f_R_ and is more related to changes in the HRV index. We conclude that the phase coherence (λ) could differ between OSA with and without MDD and between OSA patients and healthy subjects during NREM and REM sleep. Our findings suggest that the phase synchronization index between RSA and respiratory movement could provide a valuable and convenient measure for evaluating depression in OSA patients.

## Data Availability

The original contributions presented in the study are included in the article/Supplementary material, further inquiries can be directed to the corresponding author.

## Author’s note

The content of this manuscript has been presented in part at the 2022 Computing in Cardiology Conference, YA and AK (31 December 2022). Investigating Phase Coherence between Respiratory Sinus Arrhythmia and Respiration in Depressed patients with Obstructive Sleep Apnea across the Sleep Stages. 2022 Computing in Cardiology Conference. https://doi.org/10.22489/CinC.2022.327.
